# EEG-based image classification via a region-level stacked bi-directional deep learning framework

**DOI:** 10.1186/s12911-019-0967-9

**Published:** 2019-12-19

**Authors:** Ahmed Fares, Sheng-hua Zhong, Jianmin Jiang

**Affiliations:** 1 The Research Institute for Future Media Computing, College of Computer Science & Software Engineering, Shenzhen University, Shenzhen, 518060 China; 2 Department of Electrical Engineering, Computer Engineering branch, Faculty of Engineering at Shoubra, Benha University, Shoubra, Egypt; 3 National Engineering Laboratory for Big Data System Computing Technology, Shenzhen University, Shenzhen, Shenzhen, 518060 China; 4 Guangdong Key Laboratory of Intelligent Information Processing, Shenzhen University, Shenzhen, 518060 China

**Keywords:** EEG, Classification of brain activities, Region-level information, Stacked bi-directional LSTM

## Abstract

**Background:**

As a physiological signal, EEG data cannot be subjectively changed or hidden. Compared with other physiological signals, EEG signals are directly related to human cortical activities with excellent temporal resolution. After the rapid development of machine learning and artificial intelligence, the analysis and calculation of EEGs has made great progress, leading to a significant boost in performances for content understanding and pattern recognition of brain activities across the areas of both neural science and computer vision. While such an enormous advance has attracted wide range of interests among relevant research communities, EEG-based classification of brain activities evoked by images still demands efforts for further improvement with respect to its accuracy, generalization, and interpretation, yet some characters of human brains have been relatively unexplored.

**Methods:**

We propose a region-level stacked bi-directional deep learning framework for EEG-based image classification. Inspired by the hemispheric lateralization of human brains, we propose to extract additional information at regional level to strengthen and emphasize the differences between two hemispheres. The stacked bi-directional long short-term memories are used to capture the dynamic correlations hidden from both the past and the future to the current state in EEG sequences.

**Results:**

Extensive experiments are carried out and our results demonstrate the effectiveness of our proposed framework. Compared with the existing state-of-the-arts, our framework achieves outstanding performances in EEG-based classification of brain activities evoked by images. In addition, we find that the signals of Gamma band are not only useful for achieving good performances for EEG-based image classification, but also play a significant role in capturing relationships between the neural activations and the specific emotional states.

**Conclusions:**

Our proposed framework provides an improved solution for the problem that, given an image used to stimulate brain activities, we should be able to identify which class the stimuli image comes from by analyzing the EEG signals. The region-level information is extracted to preserve and emphasize the hemispheric lateralization for neural functions or cognitive processes of human brains. Further, stacked bi-directional LSTMs are used to capture the dynamic correlations hidden in EEG data. Extensive experiments on standard EEG-based image classification dataset validate that our framework outperforms the existing state-of-the-arts under various contexts and experimental setups.

## Background

In recent years, numerous noninvasive measurements of brain activities have been proposed and applied in clinical treatment and scientific research communities. One of the most popular techniques is electroencephalography (EEG). EEG is a recording of voltage fluctuations produced by ionic current flows on a variety of locations on the scalp. While reflecting the brains’ spontaneous electrical activities, EEG has the potential to provide a subjective response based on their own experiences. EEG attracts many research efforts due to its noninvasive way of measuring/acquiring brain signals and easy recording with high temporal resolution and low-cost equipment. As a result, understanding EEGs evoked by specific stimuli has been the goal for a variety of fields such as brain-computer interface (BCI) [1, 2], emotion classification [3], medical diagnosis [4–8], etc. While it is relatively easy to identify brain patterns related to audio stimuli or associated to specific diseases, it is much more difficult to understand what happens inside human brains when interacting with visual scenes [9].

The idea of reading-the-minds while performing specific tasks has been long investigated, especially for building BCIs and other EEG-related research. Most of these studies have mainly performed binary EEGs classification, including presence or absence of a specific pattern such as P300 detection [10, 11] and seizure detection [5, 12]. As known, several neurocognitive studies [13, 14] have discovered that human brain activities contain detectable patterns related to visual stimuli categories [15–17].

Unfortunately, in comparison with the success of content-based multimedia understanding over the past decades, EEG-based image classification still has a large room for improvement with respect to several evaluation criteria, including its accuracy, generalization, and interpretability.

With the extensive application and in-depth promotion of deep learning, an ever-increasing number of deep learning models are proposed for content understanding or pattern recognition of brain activities via EEGs [4, 18–31]. In these methods, the original EEG data or the extracted time-frequency features based on signal analysis algorithms are often used as the input, and some characters of human brains have not been seriously considered, such as hemispheric lateralization. Although the macrostructure of the right and left hemispheres of the human brain appears to be similar, different composition of neural networks allows for the specialized functioning in each hemisphere [32]. Hemispheric lateralization refers to the tendency for some neural functions or cognitive processes to be specialized to the right or left hemispheres of the brain. Although a growing body of evidences has suggested that cognitive tasks in human brains rely on a number of related processes whose neural loci are largely lateralized to one hemisphere or the other [33], most of current research efforts focus on studying the lateralization in different tasks [34–37], or developing better tools and models for assessing lateralization [33, 38]. Until now, no existing work for EEG-based image classification tried to integrate the hemispheric lateralization into the deep learning model to extract the region-level information from the brain signals.

Although deep learning models have been reported to achieve performance improvement for EEG-based object classification, most of these models ignore the dynamic correlations embedded inside EEGs. In those existing models, Convolutional neural networks (CNN) can extract static information from each timestamp of EEG data. Compared with CNN, unidirectional recurrent neural networks reported in [18] are capable of preserving and extracting the information from the past. But it ignores the related dynamic information from the future. As object classification is a high-level cognitive task, the electrical activations from both the past and the future have dynamic correlations with the current spontaneous response and the state of subject. To this end, it becomes desirable to consider these attributes and factors in developing next generation deep learning models for brain activity analysis and understanding.

To tackle the aforementioned challenges, we propose a region-level stacked bi-directional deep learning approach, extending [39] from single to stacked bi-directional network and conduct new analysis, comparisons, and experiments, for EEG-based image classification. By considering the hemispheric lateralization of human brains, the region-level information as the input of the deep learning model is extracted to further strengthen and emphasize the differences between two hemispheres with low dimension, and the stacked bi-directional long short-term memories (BiLSTMs) are used to capture the dynamic correlations across EEG sequences.

In comparison with the existing state-of-the-arts, our proposed model achieves a number of advantages and novelties, which can be highlighted as: (i) inspired by the hemispheric lateralization in cognitive tasks, we introduce a new concept of region-level computation into the deep learning framework to provide an alternative solution for the problem of EEG-based image classification; (ii) we propose a new deep brain analytics framework to capture the dynamic correlations across EEG sequences; and finally (iii) we carry out extensive experiments and the results demonstrate that our deep framework achieves superior performances in comparison with the existing state-of-the-arts.

The rest of the paper is organized as follows. In “Related Work” section, we present a literature survey about the existing methods that use deep learning models for EEG-based image classifications. In “Methods” section, we describe the details of our proposed region-level stacked bi-directional deep learning approach for EEG-based image classification. In “Results” section, we report our extensive experimental results and validate the superiority and effectiveness of our proposed framework, compared with the existing state-of-the-arts. In “Discussion” section, we give overall discussion,and finally “Conclusions” section provides concluding remarks and future work.

## Related Work

In general, the EEG data analysis and processing method mainly includes two steps: feature extraction and pattern recognition or machine learning-based methods to complete the signal analysis[40, 41]. Before the popularity of deep learning, the primary approaches for feature extraction mainly included time-frequency features extracted by signal analysis methods, such as power spectral density [42], bandpower [43], independent components [44], and differential entropy [45]. The widely researched pattern recognition and machine learning methods include artificial neural networks [46, 47], naive Bayes [48], support vector machines (SVM) [49, 50], etc. With the extensive application and in-depth promotion of deep learning, an ever-increasing number of brain science and neuroscience research teams are exploiting its strength in designing algorithms to achieve intelligent understanding and analysis of brain activities via EEGs, leading to propose an end-to-end model by integrating feature extraction and classification/clustering.

Jiao et al. [23] proposed a multi-channel deep convolution network to classify mental loads. Wang et al. [24] used LSTM network to classify motor imagery tasks, and used a one-dimensional aggregation approximation method to extract the network’s effective features.

Cole et al. [25] used a predictive modelling approach based on CNN for predicting brain ages. Their analysis showed that the brain-predicted age is highly reliable. Gao et al. [26] proposed a spatiotemporal deep convolution model, which significantly improved the accuracy of detecting driver fatigue by emphasizing the importance of spatial information and time dependence of EEGs. Yuan et al. [27] proposed an end-to-end multi-view deep learning framework to automatically detect epileptic seizures in EEG signals. Li et al. [28] tried to incorporate transfer learning into the construction of convolutional neural networks and successfully applied the model to the clinical diagnosis of mild depression. Dong et al. [20] used a rectified linear unit (ReLU) activation function and a mixed neural network of LSTM on time-frequency-domain features to classify sleep stages. Lawhern et al. [29] proposed a compact full convolutional network as the EEG-specific model (EEGNet) and applied it to four different brain-machine interface classification tasks. Zhang et al. [30] proposed a cascaded and parallel convolution recurrent neural network model to accurately identify human expected motion instructions by effectively learning the spatio-temporal representation of the original EEG signal. Tan et al. [31] converted EEG data into EEG-based video and optical flow information, classified them by CNN and RNN, and established an effective rehabilitation support system based on BCI.

Multimedia data, which contain a large amount of content information and rich visual characteristics, are considered to be a very suitable stimuli material and widely used in the acquisition and analysis of EEG signals [9, 18, 51]. Researchers tried to identify and classify the content information of multimedia data viewed by users through the analysis of EEG signals [15, 52, 53]. Spampinato et al. [18], used LSTM network to learn an EEG data representation based on image stimuli and constructed a mapping relationship from natural image features to EEG representation. Finally, they used the new representation of EEG signals for classification of natural images. Compared with traditional methods, these deep learning-based approaches have achieved outstanding classification results.

Recent studies have shown that it is possible to reconstruct multimedia content information itself by mining EEG data. Kavasidis et al. [9] proposed a method for reconstructing visual stimuli content information through EEGs. By using a variable-valued autoencoder (VAE) and generative adversarial networks (GANs), they found that EEG data contain patterns related to visual content, and the content can be used to generate images that are semantically consistent with the input visual stimuli. While these methods have demonstrated the capability of using deep learning framework for EEG-based image classification, the original EEG data or the extracted time-frequency features based on signal analysis algorithms are often used as the input, and some characteristics of human brains have not been seriously considered, such as hemispheric lateralization, and the classification accuracy achieved to date by Spampinato et al. was 82.9*%* [18], leaving significant space for further research and improvement.

## Methods

**Fig. 1:**
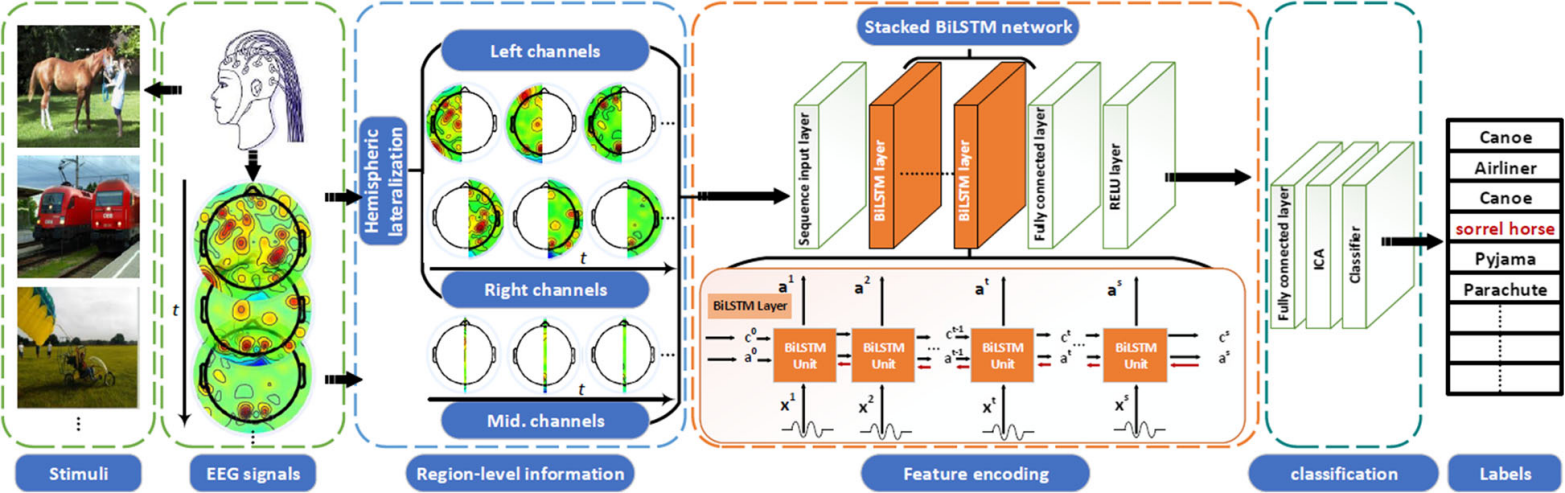
Structural illustration of the proposed deep framework

Given the extensive survey on existing research in the previous section, we propose a novel region-level stacked bi-directional deep learning framework for visual object classification. Our approach consists of three stages, including the region-level information extraction stage, the feature encoding stage and the classification stage. The structural illustration is given in Fig. 1.

### The region-level information extraction stage

Although a growing body of evidence has suggested that in some cognitive processes, the neural loci are largely lateralized to one hemisphere or the other, no existing work for EEG-based image classification tried to integrate this concept into the deep learning model to extract the region-level information from the brain data. In this section, we seek to extract region-level information from the raw EEG signals. For channel *i*, the raw EEG signal denoted as **s**_*i*_ is considered as input to the region-level information extraction stage, where *i*∈[1, *l*_*ch*_=128] is the index for channels, and *l*_*ch*_ is the number of channels. Following that, the region-level information extraction stage splits the EEG data into three groups, including the left hemisphere, the right hemisphere, and the middle group. Denoting the left hemisphere group, the right hemisphere group, and the middle group as, **S**^[*l*]^, **S**^[*r*]^, and **S**^[*m*]^, respectively, we attach each channel **s**_*i*_ to one group based on their corresponding electrode physical location. Each channel in the left hemisphere group has a corresponding channel in the right hemisphere group, and hence, the difference, **d**_*j*_, can be calculated according to the following equation:

1 $$ \mathbf{d}_{j} = \mathbf{S}_{j}^{[l]} - \mathbf{S}_{j}^{[r]}  $$

where $\left (\mathbf {S}_{j}^{[l]},\ \mathbf {S}_{j}^{[r]}\right)$ is considered as the corresponding pair. *j*∈[1, *l*_*g*_] is the index for the left hemisphere, the right hemisphere, and the difference, and *l*_*g*_ is the number of channels attached to the left hemisphere or the right hemisphere. The output of the region-level information extraction stage is obtained when the difference, $\mathbf {D}={\left [ \mathbf {d}_{j} \right ]}_{j=1}^{l_{g}}$, is combined with the middle hemisphere group, **S**^[*m*]^, into one variable, **X**, and pass it to the feature encoding stage as an input according to the following equation:

2 $$ \mathbf{X}=\left[{\mathbf{D}}^{{\mathtt{T}}}\ {\mathbf{S}^{[m]}}^{\mathtt{{T}}}\right]  $$

### The feature encoding stage

The feature encoding stage aims at extracting the EEG description from the region-level information via a stacked bi-directional LSTM network. The bi-directional LSTM learns long-term dependencies between time steps of the sequence data. It not only solves the vanishing gradient problem, which appears in recurrent neural network (RNN) through the forget gate *Γ*_*f*_ and the update gate *Γ*_*u*_, but also captures the dynamic correlations inside the EEG sequences. In contrast to the unidirectional LSTM, the bi-directional LSTM calculates the output **y**^*t*^ at any point of time *t* by taking information from both earlier output state $\overrightarrow {\mathbf {a}}^{t}$ and later output state $\overleftarrow {\mathbf {a}}^{t}$ in the sequence, as shown in Eq. (3).

3 $$ \mathbf{y}^{t} = \sigma_{y}\left(\mathbf{W}_{y}\left[\overrightarrow{\mathbf{a}}^{t}, \overleftarrow{\mathbf{a}}^{t}\right] + \mathbf{b}_{y}\right)   $$

**Fig. 2:**
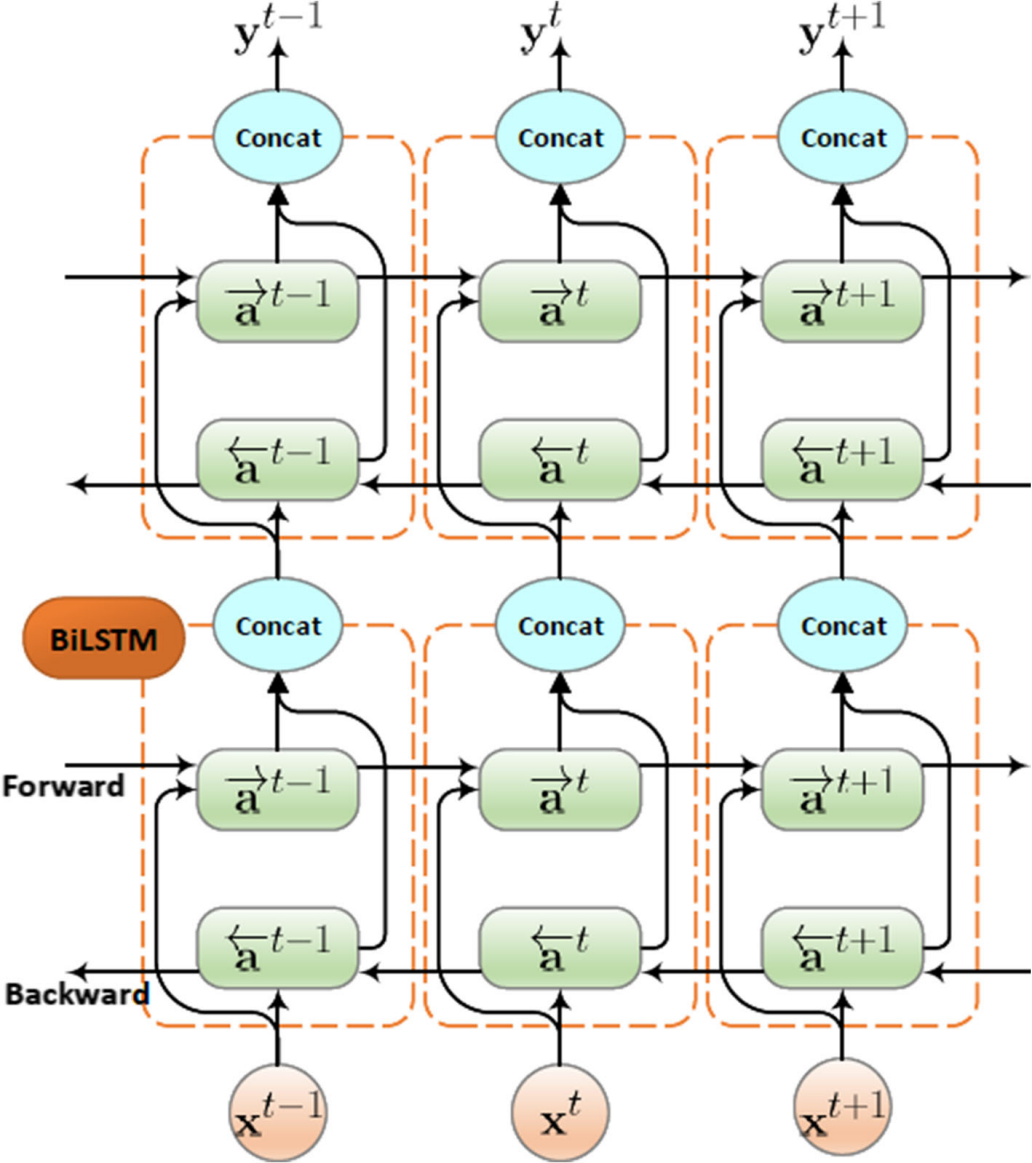
Two stacked layer structure in bi-directional LSTM with three consecutive time steps

**Fig. 3:**
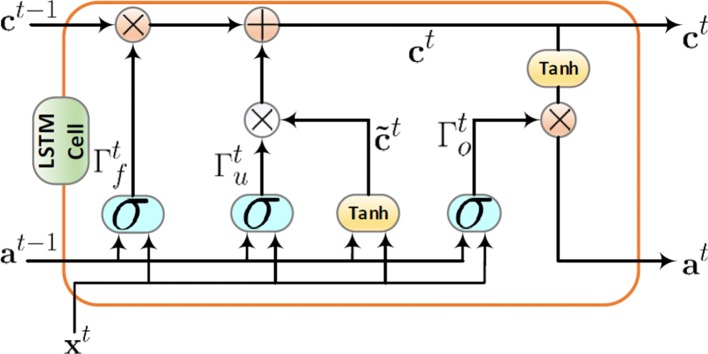
Long short-term Memory cell

The encoder network is constructed by a stack of *v* bi-directional LSTM layers as illustrated in Fig. 2. At each time step *t*, the first bi-directional LSTM layer takes the input as the region-level information output, $\mathbf {X}= {\left [ \mathbf {x}^{t} \right ]}_{t=1}^{l_{s}}$, where *l*_*s*_ is the length of sequence. If other bi-directional LSTM layers are present, the output of the first layer is provided as input to the second layer and so on. The ouput of the deepest bi-directional LSTM layer at the last time step is used as the EEG decription for the whole region-level information sequence. The structure of the layer in bi-directional LSTM, containing a forward layer and a backward layer, is illustrated in Fig. 2. As seen, the forward layer output sequence, $\overrightarrow {\mathbf {a}}^{t}$, is iteratively calculated using inputs in a sequence from time 1 to time *t*−1, while the backward layer output sequence, $\overleftarrow {\mathbf {a}}^{t}$, is calculated using the inputs from the end of sequence to time *t*+1. Both the forward and backward layer outputs are calculated by using the standard LSTM updating equations [54]. The LSTM uses custom-built memory cells to store information, and these memory cells are used in finding and exploiting long range dependencies. Figure 3 shows a single LSTM memory cell. At each time *t*, the LSTM takes the layer input **x**^*t*^, the previous layer output **a**^*t*−1^, and the previous cell output state **c**^*t*−1^ as its inputs, and produces the layer output **a**^*t*^ and the cell output state **c**^*t*^ as the its outputs. The memory cell also takes into account the candidate for replacing the memory cell, $\tilde {\mathbf {c}}^{t}$, while training and updating parameters. There are three gates in an LSTM cell, including an update gate, *Γ*_*u*_, a forget gate, *Γ*_*f*_, and an output gate, *Γ*_*o*_. According to the gated structure, an LSTM can manage long-term dependencies to allow useful information pass through the network. At time *t*, for example, the update gate $\Gamma _{u}^{t}$, the forget gate $\Gamma _{f}^{t}$, the output gate $\Gamma _{o}^{t}$, and the candidate for replacing the memory cell $\tilde {\mathbf {c}}^{t}$, can be calculated according to the following equations:

4 $$ \Gamma_{f}^{t} = \sigma\left(\mathbf{U}_{f} \mathbf{a}^{t-1}+ \mathbf{W}_{f} \mathbf{x}^{t} + \mathbf{b}_{f}\right)   $$

5 $$ \Gamma_{u}^{t} = \sigma\left(\mathbf{U}_{u} \mathbf{a}^{t-1}+ \mathbf{W}_{u} \mathbf{x}^{t} + \mathbf{b}_{u}\right)   $$

6 $$ \Gamma_{o}^{t}= \sigma\left(\mathbf{U}_{o} \mathbf{a}^{t-1}+ \mathbf{W}_{o} \mathbf{x}^{t} + \mathbf{b}_{o}\right)   $$

7 $$ \tilde{\mathbf{c}}^{t} = \tanh\left(\mathbf{U}_{c} \mathbf{a}^{t-1}+ \mathbf{W}_{c} \mathbf{x}^{t} + \mathbf{b}_{c}\right)   $$

where, for *k*∈{*f*,*u*,*o*,*c*}, **W**_*k*_ is the weight matrix mapping the layer input (the region-level information, $\mathbf {X}= {\left [ \mathbf {x}^{t} \right ]}_{t=1}^{l_{s}}$) to the three gates and the candidate for replacing the memory cell. While **U**_*k*_ is the weight matrix connecting the previous cell output state to the three gates and the candidate for replacing the memory cell, **b**_*k*_ is the bias vector. The functions *σ*() and tanh() are the element-wise sigmoid and hyperbolic tangent, respectively.

Based on the results of the Eqs. 4-(7), at each time iteration *t*, the cell output state **c**^*t*^, and the layer output **a**^*t*^, can be calculated according to the following equation:

8 $$ \mathbf{c}^{t} = \Gamma_{f}^{t}\times \mathbf{c}^{t-1} + \Gamma_{u}^{t}\times \tilde{\mathbf{c}}^{t}   $$

9 $$ \mathbf{a}^{t} = \Gamma_{o}^{t}\times \tanh\left(\mathbf{c}^{t}\right)  $$

The final output of an LSTM layer is a vector of all outputs, represented by $\mathbf {Y}={\left [ \mathbf {y}^{t}\right ]}_{t=1}^{l_{s}}$, at any time step **y**^*t*^, which can be calculated according to Eq. 3. When taking the EEG-based image classification as an example, only the last element of the output vector, $\phantom {\dot {i}\!}\mathbf {y}^{l_{s}}$, is considered.

### The classification stage

The classification stage consists of an independent component analysis (ICA) module and a classifier layer. The ICA is placed before the layer of classifiers as a feature selection module, which takes the EEG description from the stacked bi-directional LSTMs network as an input and returns the independent statistical features as an output. Two classifiers have been investigated in this paper, including the SoftMax classifier and the multiclass support vector machine (SVM).

## Results

To evaluate our proposed region-level stacked bi-directional deep learning framework, we conduct three phases of experiments. In the first phase, we evaluate the classification performance of our proposed deep learning framework on the largest standard dataset for EEG-based image classification: ImageNet-EEG [18]. In the second phase, we try to study the classification performance of the proposed framework upon different EEG frequency bands, including Beta and Gamma bands. In the third phase, we study the relationships between the neural activations and the specific emotional states.

### Experimental Settings

All the experiments are conducted on the standard dataset for EEG-based image classification: ImageNet-EEG. This dataset is a publicly available EEG dataset for brain imaging classification proposed by Spampinato et al. [18]. ImageNet-EEG is collected using a 128-channel cap with active, low-impedance electrodes (actiCAP 128Ch). It includes the EEG signals of six subjects produced by asking them to look at the visual stimuli, which are images selected from a subset of ImageNet [55], containing 40 classes and each class has 50 images. During the subjective experiment, each image was shown on the computer screen for 500 ms. The sampling frequency and data resolution were set to 1kHz and 16 bits, respectively. For benchmarking purposes, the proposed framework is compared with the EEG-based image classification methods [18, 56], which are the latest deep learning methods on the same dataset, and the baseline method: representational similarity based Linear discriminant analysis (RS-LDA) [57].

For our method, the iteration limit is set as 2500, and the batch size is 440 for the feature encoding stage of the stacked bi-directional LSTMs. There are two layers in the stacked bi-directional LSTM network (*v*=2), and number of nodes in each layer is 68. Concerning the parameters for ICA, the number of extracted features is 60, and the iteration limit is set to 400. Our framework is implemented on the Tesla ^*Ⓡ*^ P100 GPU.

### EEG-Based image Classification

In the first phase of experiments, we try to validate the effectiveness of our region-level stacked bi-directional deep learning framework for EEG-based image classification. All the experimental setting follows that of the existing work [18].

Table 1 provides the experimental results in terms of the classification accuracies for our proposed framework, the existing state-of-the-art RNN-based method [18], siamese network [56], and the RS-LDA method [57]. As seen, while the precision rate achieved by our proposed region-level stacked bi-directional deep learning framework is 97.3*%*, the existing state-of-the-art, siamese network, and the RS-LDA compared are 82.9*%*, 93.7*%* and 13.0*%*, respectively.

**Table 1:** The classification performance comparisons among our proposed framework, RNN-based, siamese network, and the RS-LDA

Models	Accuracy
Proposed region-level stacked bi-directional LSTMs	97.3*%*
Siamese network [56]	93.7*%*
RNN-based model [18]	82.9*%*
RS-LDA [57]	13.0*%*

### Study of Different Frequency Bands

To test our proposed region-level stacked bi-directional deep learning framework upon different frequancy bands, we carry out the second phase of experiments on the same dataset ImageNet-EEG [18]. The EEG data in ImageNet-EEG [18] has been filtered by a notch filter (49-51 Hz) and a second-order band-pass Butterworth filter (low cut-off frequency 14 Hz, high cut-off frequency 71 Hz). Therefore, the recorded signal only included the Beta (15-31 Hz) and Gamma (32-70 Hz) rhythm bands. Beta wave is seen usually on both sides in symmetrical distribution and is most evident frontally. It is closely associated with normal waking consciousness. As known, low amplitude beta with multiple and varying frequencies is often associated with active, busy or anxious thinking and active concentration [58]. Gamma band is used to represent binding of different populations of neurons together into a network for the purpose of carrying out a certain cognitive or motor function [59]. Here, we conduct a detailed examination to study the contributions from each frequency band.

Table 2 summarizes the experimental results obtained by the proposed framework across different EEG frequency bands. Here, we do not change the deep learning framework described before. We just extract the signal in the same format but with the specific frequency band as the input for our framework. From Table 2, it can be seen that the classification accuracy achieved by the signal only with Gamma band is close to the best accuracy (97.3*%*), and better than that of the signal only with Beta band. While the classification accuracy achieved by the signal only with Beta band is 94.90*%*, the classification accuracy achieved by the signal only with Gamma band is 96.89*%*. The classification results are consistent with the discoveries of the existing work, demonstrating that synchronization of neural activity in the Gamma band plays a significant role in the classification of objects or other related visual perceptions or higher cognitive functions [60].

**Table 2:** The Classification accuracies achieved by different rhythm band

Input Rhythm Band	Accuracy
Beta band only	94.90%
Gamma band only	96.89%

### Case study of the neural activations and emotions

Different from most of the existing EEG datasets that only include less than 10 categories, ImageNet-EEG contains 40 categories, and most of them are common objects or animals. Hence, for this dataset, we are not satisfied in just presenting the novel deep learning framework with good classification results. In the third phase of experiments, we try to study the relationships between the neural activations and the specific emotional states. The EEG data in ImageNet-EEG only contains the Beta and Gamma bands. From the existing work, the emotional processing enhanced Gamma band powers at frontal area as compared to processing neutral pictures [61], and the signal from Gamma band is suitable for EEG-based emotion classification [62]. Thus, all experiments provided here are focused on the signals from Gamma band.

**Fig. 4:**
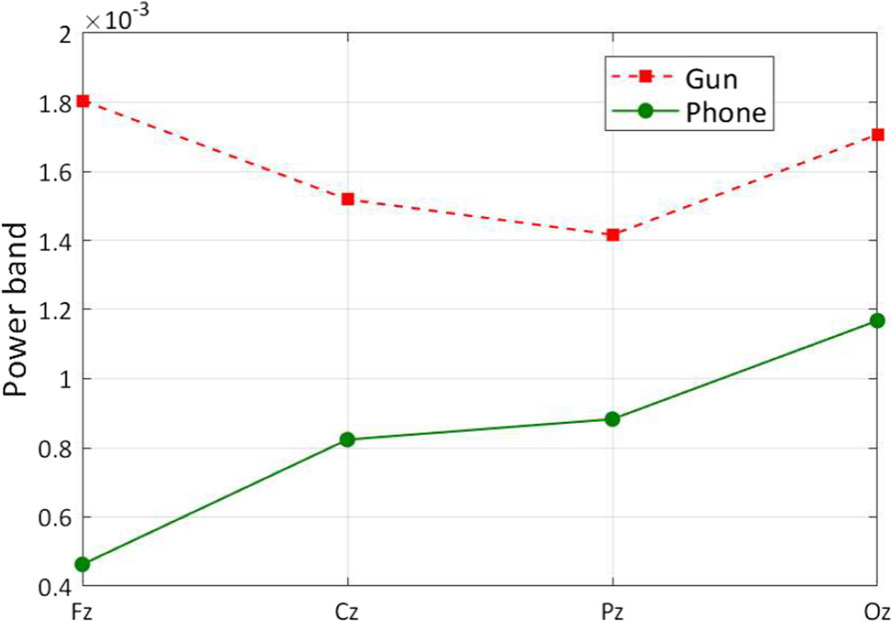
The average power in Fz, Cz, Pz, and Oz locations when stimuli are from the categories of “gun” and “phone”, respectively

This dataset includes 40 categories, including “dog”, “cat”, “butterfly”, “sorrel”, “capuchin”, “elephant”, “panda”, “fish”, “airliner”, “broom”, “canoe”, “phone”, “mug”, “convertible”, “computer”, “watch”, “guitar”, “locomotive”, “espresso”, “chair”, “golf”, “piano”, “iron”, “jack”, “mailbag”, “missile”, “mitten”, “bike”, “tent”, “pajama”, “parachute”, “pool”, “radio”, “camera”, “gun”, “shoe”, “banana”, “pizza”, “daisy” and “bolete” (fungus). Each category contains 50 images with 300 EEG signals for the six subjects. In these categories, the class “gun” is a category that could obviously cause negative emotions. Most of other categories are thought as the typical neutral ones, such as “phone”, “watch”, “bike”, “shoe”. We calculate the average power of the EEG data from the classes “gun” and “phone” in different locations. The experimental results are provided in Fig. 4. From these results, we can find the EEG data with the stimuli from the negative category “gun” contain a higher power in Fz. It means that, compared with the central, parietal and occipital areas, the negative emotional processing enhances Gamma band power at frontal areas.

**Fig. 5:**
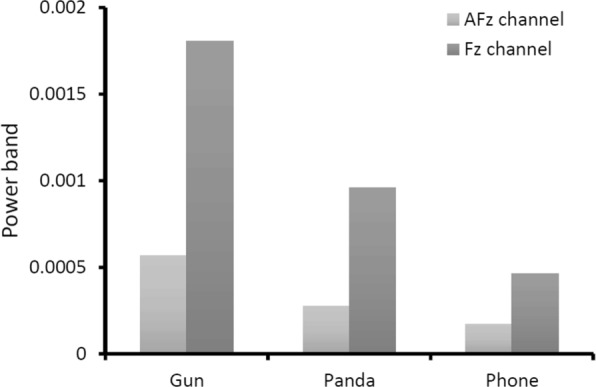
The average power of the AFz and Fz locations for the categories of “gun”, “panda”, and “phone”

We compare the average power of the EEG data from the classes “gun”, “panda”, and “phone” in AFz and Fz in Fig. 5. The giant panda is easily recognized by the large, distinctive black patches around its eyes, over the ears, and across its round body. It is thought as one of the world’s most adored and protected rare animals. Hence, we estimate it may trigger positive emotion compared with other categories in ImageNet-EEG. From the experimental results, it is validated that there exists a statistically significant effect as such that the neural patterns for the category “gun” have higher gamma responses at prefrontal sites than the category “panda” and “phone”, and this result is consistent with the findings that the neural patterns have higher gamma responses at prefrontal sites for negative emotions [63].

**Fig. 6:**
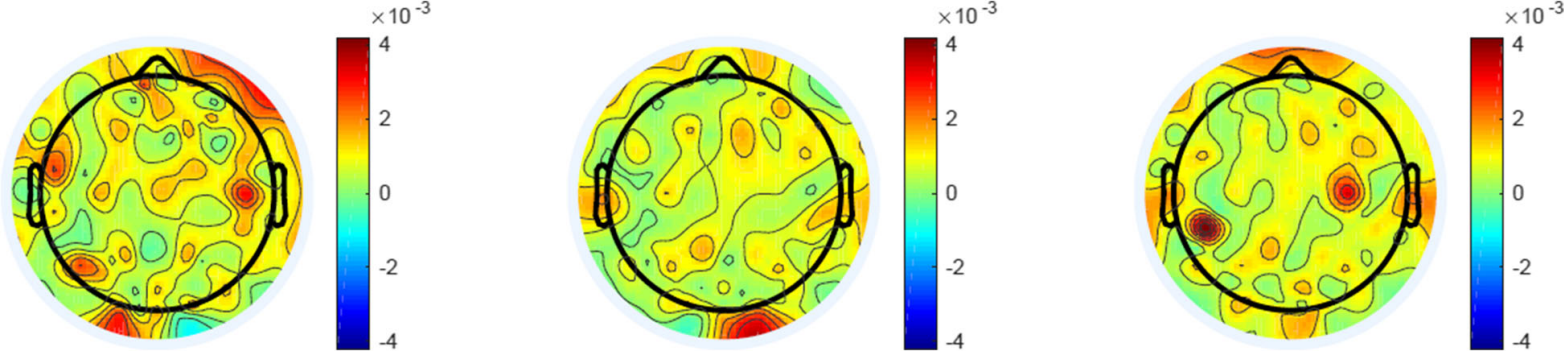
Scalp distribution of the average energy at Gamma frequency sub-band for all participants and sessions of the three categories: “gun”, “phone”, and “panda”

Figure 6 demonstrates the average energy distribution in Gamma band of the “gun”, “phone”, and “panda” categories. From this figure we can see that an increase of the average relative energy in the prefrontal area during the period of the images from the category “gun” is observed as compared to that from the category “phone” and “panda”. These results are consistent with our previous observations in Fig. 4, Fig. 5, and some existing work [64, 65], which shows that neural signatures associated with positive, neutral and negative emotions do exist.

## Discussion

Study on human brain intelligence has been researched across a number of areas, including neuroscience, brain science, and computer science, in which EEG-based interfacing with brains remains one of the most popular methods [10, 15, 66]. While artificial intelligence is becoming the most actively pursued topic in computer vision, exploitation of brain intelligence could provide enormous potential for further advancing AI techniques as well as their practical applications. In CVPR2017, Spampinato et al. [18] reported their work on EEG-based brain recognition of visual object categories via deep learning and achieved significantly improved results. As their deep learning model is primarily limited to the existing approaches, however, there still exist enormous spaces for further research, especially in terms of exploiting the unique feature of brain intelligence.

To exploit the features of brain intelligence and achieve further improvement upon the deep learning based brain recognition of visual object categories, we introduce a new concept of integrated hemispheric lateralization stacked bi-directional deep learning, where the region-level information, as the input of the stacked deep learning model, is extracted to further strengthen and emphasize the differences between two hemispheres and this leads to improve the recognition performances.

To future discuss the contribution of each stage designed in our proposed region-level stacked bi-directional deep learning framework, we further run experiments to explore the effectiveness of different configurations made by individual stages. In the first stage, individual elements considered, including with or without the region-level information. In the second stage, we provide the results with three different feature-encoding techniques, including unidirectional LSTM, bi-directional LSTM, and stacked bi-directional LSTMs. In the third stage, we provide the results with different classifiers, including SoftMax and SVM.

Table 3 reports the experimental results in classification precision rates with all the various configurations, from which we can observe and draw a number of conclusions that can be described as follows.

**Table 3:** Comparative assessment of the proposed framework upon different configurations

	Framework					
Configurations	1	2	3	4	5	6
Lateralization	Region-level info.	Region-level info.	None	Region-level info.	Region-level info.	Region-level info.
Feature encoder	Unidirectional LSTM	Unidirectional LSTM	BiLSTM	BiLSTM	BiLSTM	Stacked biLSTM
Classifier	SoftMax	ICA+SVM	SoftMax	SoftMax	ICA+SVM	ICA+SVM
Accuracy	92.9%	94.3%	95.3%	97.0%	97.1%	97.3%

(i) The performance of bi-directional LSTM is always better than that of the unidirectional LSTM as the feature encoder in the second stage. These results are demonstrated by configurations 1 to 5 in Table 3. While the best performance with the unidirectional LSTM is 94.3*%* (configuration 2), the best performance with the bi-directional LSTM is 97.1*%* (configuration 5).

(ii) The performance of using the region-level information is better than of without using the the region-level information in the first stage. These results are demonstrated by configurations 3 to 5 in Table 3. While the best performance of without using the region-level information is 95.3*%* (configuration 3), the best performance of using the region-level information is 97.1*%* (configuration 5).

(iii) The performance using ICA plus SVM is always better than that of SoftMax in the second stage. These results are demonstrated by configurations 1 to 6 in Table 3. If the unidirectional LSTM is selected as the feature encoder, the best performance achieved with SoftMax is 92.9*%* (configuration 1), and the best performance achieved by SVM classifier is 94.3*%* (configuration 2). A similar case happens when the bi-directional LSTM is selected as the feature encoder.

(iv) The performance of the stacked bi-directional LSTMs is better than that of the unidirectional LSTM and bi-directional LSTM as the feature encoder in the second stage. These results are demonstrated by configurations 1 to 6 in Table 3. While the best performance with the unidirectional LSTM and bi-directional LSTM are 94.3*%* and 97.1*%*, respectively (configuration 2 and configuration 5), the best performance with the stacked bi-directional LSTMs is 97.3*%* (configuration 6).

## Conclusions

In this research, by combining region-level information and stacked bi-directional LSTMs together into a complete system, we propose a novel architecture for EEG-based image classification. To the best of our knowledge, such attempts have never been reported in the literature before, indicating a certain level of novelties. In our novel architecture, the region-level information is extracted to preserve and emphasize the hemispheric lateralization for neural functions or cognitive processes inside human brains. Further, stacked bi-directional LSTMs are used to capture the dynamic correlations hidden in EEG data. Extensive experiments are conducted on standard EEG-based image classification dataset ImageNet-EEG, in order to assess the accuracy of the proposed framework and validate that our framework outperforms the existing state-of-the-arts under various contexts and experimental setups. We also find that the signal of Gamma band is useful to achieve good performances in the classification of objects, and it also plays a significant role in the classification of emotions, which validate that neural signatures associated with positive, neutral and negative emotions do exist. Further, our research has produced substantial evidences to support that data estimated straightforwardly from human minds could enable machine learning models to make better and more human-like judgements.

Two possibilities can be identified for further research, which include: (i) applying our deep learning framework for other EEG-based content understanding or pattern analysis tasks; (ii) reconstructing the multimedia content information through the proposed EEG representations.

## Data Availability

Data used for this study is from Pattern Recognition and Computer Vision Laboratory, the data is a publicly available EEG dataset for brain imaging classification hosted by http://perceive.dieei.unict.it.

## Abbreviations

BCI: Brain-computer interface
BiLSTMs: Bi-directional long short-term memories
CNN: Convolutional neural network
EEG: Electroencephalogram
GANs: Generative adversarial networks
ICA: Independent component analysis
ReLU: Rectified linear unit
RNN: Recurrent neural network
RS-LDA: Representational similarity based Linear discriminant analysis
SVM: Support vector machines
VAE: Variable-valued autoencoder
